# Molecular identification and characterization of *Wolbachia* and *Cardinium* with co-occurrence of *Leishmania* spp. in *Culicoides* biting midges (Diptera: Ceratopogonidae) from leishmaniasis-affected areas of Thailand

**DOI:** 10.1016/j.crpvbd.2026.100387

**Published:** 2026-05-16

**Authors:** Sakone Sunantaraporn, Pathamet Khositharattanakool, Puckavadee Somwang, Pranyu Leemingsawat, Picha Pattrapruettada, Darlene Ariyaskul, Tinn Hongboontry, Chitchanok Cherdchoochart, Rungfar Boonserm, Thanapat Pataradool, Padet Siriyasatien

**Affiliations:** a Center of Excellence in Vector Biology and Vector-Borne Diseases, Department of Parasitology, Faculty of Medicine, Chulalongkorn University, Bangkok, 10330, Thailand; b School of Medicine, Mae Fah Luang University, Chiang Rai, 57100, Thailand; c Biomedical Technology Research Group for Vulnerable Populations, Mae Fah Luang University, Chiang Rai, 57100, Thailand; d Department of Parasitology, Faculty of Medicine, Chulalongkorn University, Bangkok, 10330, Thailand; e International Medical Program, Faculty of Medicine, Chulalongkorn University, Bangkok, 10330, Thailand

**Keywords:** *Culicoides*, *Leishmania*, *Wolbachia*, *Cardinium*, Thailand

## Abstract

Biting midges of the genus *Culicoides* have been identified as potential vectors for the transmission of species of the *Leishmania* subgenus *Mundinia*, the causative agents of autochthonous leishmaniasis in Thailand. Vector competence may be potentially affected by bacterial endosymbionts; however, there is no known correlation between these endosymbionts and *Leishmania* parasites in *Culicoides* biting midges. In this study, we aimed to explore the prevalence and association of bacterial endosymbionts and the detection of *Leishmania* DNA in *Culicoides* spp. Female midges were captured at five sampling sites in areas with autochthonous leishmaniasis in northern and southern Thailand. *Culicoides* species were identified using both morphological characteristics and *cox*1 sequencing. The presence of *Wolbachia*, *Cardinium*, and *Leishmania* DNA in individual midges was molecularly screened targeting the *wsp* and 16S rRNA genes, and the ITS1 region, respectively. All amplification products were sequenced and subjected to phylogenetic analysis. A total of 593 female midges were collected, comprising 21 species of *Culicoides* and one species of *Culicoides* (*Trithecoides*). The *Wolbachia* isolates from infected *Culicoides* spp. were phylogenetically classified into supergroups A, B, and F. Six *Wolbachia* putative strains belonged to clade Wol-b (Wol-b1 to Wol-b6), two to clade Wol-a (Wol-a1 and Wol-a2), and one to clade Wol-f (Wol-f1). Three *Wolbachia* strains were identified as *w*Kerlac, *w*Beva_B, and *w*CauA. Moreover, both *Cardinium* groups A and C were identified. It is noteworthy that co-infections between bacterial endosymbionts and *Leishmania* spp. showed a significant association. To the best of our knowledge, this study provides the first evidence of *Wolbachia* and *Cardinium* in *Culicoides* spp. from leishmaniasis-affected areas in Thailand. Detecting bacterial endosymbionts co-occurring with *Leishmania* spp. in *Culicoides* biting midges may suggest a potential, but unconfirmed, antagonistic effect on *Leishmania*. This provides preliminary data that could inform the development of new vector control strategies for diseases transmitted by *Culicoides* spp. in Thailand.

## Highlights

• First report of *Wolbachia* and *Cardinium* endosymbionts in *Culicoides* spp. from leishmaniasis-affected areas in Thailand.
• The prevalence of *Wolbachia*, *Cardinium*, and co-infection in *Culicoides* was 29.0%, 14.5%, and 11.47%, respectively.
• *Wolbachia* supergroups A, B, and F were identified in *Culicoides* biting midges.
• Three *Wolbachia* putative strains were characterized as *w*Kerlac, *w*Beva_B, and *w*CauA.
• *Cardinium* groups A and C were identified in *Culicoides* biting midges.

## Graphical abstract

**Figure:**
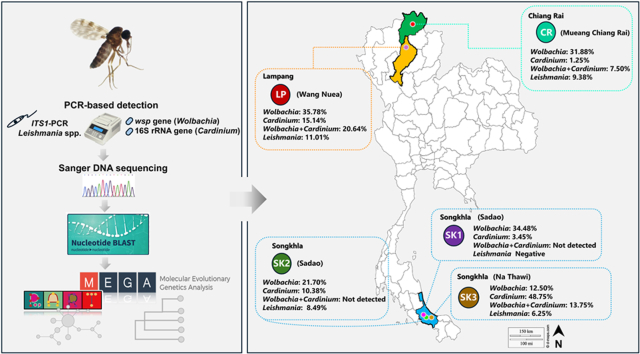

## 1 Introduction

Leishmaniasis is a neglected tropical disease caused by obligate intracellular parasites of the genus *Leishmania*. In Thailand, more than 20 cases of autochthonous leishmaniasis have been documented in both immunocompromised and immunocompetent individuals across the northern and southern regions of the country. The causative agents of autochthonous leishmaniasis in Thailand are *Leishmania martiniquensis* (Chiewchanvit et al., 2015; Phadungsaksawasdi et al., 2026; Rattanagitpaisan et al., 2026) and *Leishmania orientalis* (Jariyapan et al., 2018), which have been phylogenetically classified within the subgenus *Mundinia* (Espinosa et al., 2018). Several studies have reported the detection of *Leishmania* DNA in phlebotomine sand flies in Thailand. For example, DNA of *L. martiniquensis* has been detected in several species of sand flies, including *Segentomyia gemmae*, *Se. barraudi*, *Se. khawi*, *Phlebotomus stantoni*, and *Grassomyia indica* (Chusri et al., 2014; Sriwongpan et al., 2021; Preativatanyou et al., 2023; Phumee et al., 2024). Furthermore, DNA of *L. orientalis* was identified in *Se. iyegari*, *Se. gammae*, and *Se. khawi* (Siripattanapipong et al., 2018; Sriwongpan et al., 2021; Phumee et al., 2024). However, non-sand fly vectors, such as biting midges of the genus *Culicoides* and *Forcipomyia*, are currently considered possible vectors for the transmission of *Leishmania* (*Mundinia*) species (Chanmol et al., 2019; Panahi et al., 2020; Sunantaraporn et al., 2021; Preativatanyou et al., 2024; Tepboonrueng et al., 2025). Recent investigations have successfully demonstrated the development and transmission of species of *Leishmania* (*Mundinia*), including *L. martiniquensis*, *L. orientalis*, and *L. chancei* (formerly named *Leishmania* sp. from Ghana), in *C. sonorensis* midges (Becvar et al., 2021).

Biting midges of the genus *Culicoides* are hematophagous insects belonging to the family Ceratopogonidae. It is established that *Culicoides*-borne pathogens are responsible for significant veterinary and medical diseases, including bluetongue virus (BTV), Schmallenberg virus (SBV), African horse sickness virus (AHSV), epizootic hemorrhagic disease virus (EHDV), and Oropouche virus (OROV) (Sick et al., 2019). A recent study by Fujisawa et al. (2021) demonstrated the presence of bluetongue virus in four species of *Culicoides*, namely *C. orientalis*, *C. imicola*, *C. oxstoma*, and *C. fulvus*, collected from ruminant farms in western Thailand. Furthermore, haemosporidian infection in Thai *Culicoides* biting midges has been reported by Pramual et al. (2021a) and Sunantaraporn et al. (2022). As previously stated, *Culicoides* spp. can serve as potential vectors of several pathogens for transmission to humans and/or animals.

In recent years, bacterial endosymbionts have received increasing attention for use as an insect vector control strategy, especially to control vector-borne diseases (VBDs) (Floate et al., 2006). Previous studies have shown that *Wolbachia* endosymbionts (class Alphaproteobacteria) are present in a wide range of insects and filarial nematodes worldwide. *Wolbachia* strains are well characterized in mosquitoes, particularly within the family Culicidae. In mosquitoes, *Wolbachia* infection can inhibit the replication of arboviruses, including dengue virus (DENV), chikungunya virus (CHIKV), yellow fever virus (YFV), Zika virus (ZIKV), and West Nile virus (WNV) (Ant et al., 2023), as well as *Plasmodium* parasites (Qu and Childs, 2025) and filarial nematodes (Setegn et al., 2024). *Wolbachia* infection can also cause a wide range of abnormal reproductive phenotypes through parthenogenesis, feminization, male killing, and cytoplasmic incompatibility (CI) (Stouthamer et al., 1999). To date, *Culicoides* midges have been demonstrated to have endosymbiotic bacteria of the genera *Wolbachia* and *Cardinium*. A previous study reported evidence of infection by the endosymbiont “*Candidatus* Cardinium hertigii” (Bacteroidetes group) in various *Culicoides* species and suggested the existence of a phylogenetically distinct lineage within the *Cardinium* endosymbiont group (Group C) (Noel and Atibalentja, 2006; Nakamura et al., 2009; Konecka and Olszanowski, 2019). The biological mechanisms of *Cardinium* bacteria in biting midges are uncertain, while information from different host species suggests that *Cardinium*-induced reproductive manipulations include parthenogenesis, feminization, and CI (Zchori-Fein et al., 2004; Tarlachkov et al., 2023). However, no studies have investigated the association of *Wolbachia* and *Cardinium* endosymbionts in Thai *Culicoides* species, particularly in leishmaniasis-affected areas.

The present study aimed to investigate the prevalence of *Wolbachia* and *Cardinium* endosymbiotic bacteria in field-caught *Culicoides* spp. collected from five different leishmaniasis-affected areas in northern and southern Thailand. This study provides molecular evidence of the prevalence of *Leishmania* parasites in *Culicoides* spp. and examines their association with endosymbiotic bacteria. This information would be valuable for the development of a potentially effective vector control strategy utilizing bacterial endosymbionts in the future.

## 2 Materials and methods

### 2.1 Sampling locations and biting midge trapping

**Fig. 1:**
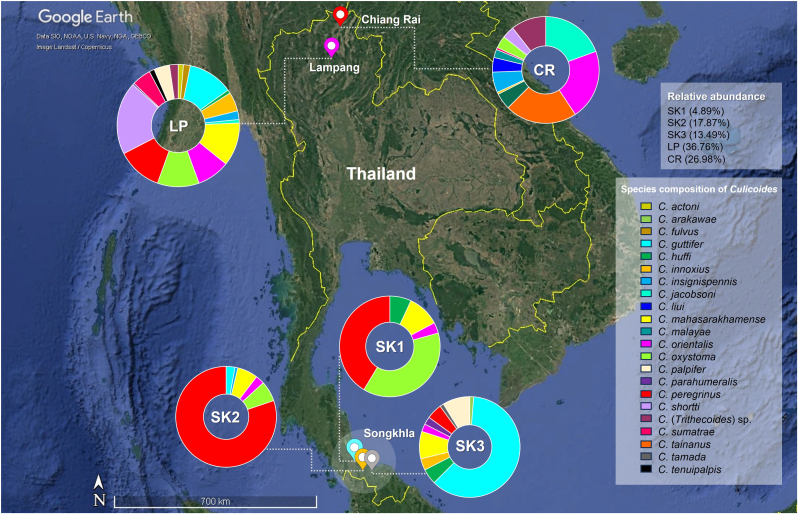
Map of Thailand showing species diversity of *Culicoides* spp. and their relative abundance at the collection sites of five autochthonous leishmaniasis-affected areas in Chiang Rai (CR), Lampang (LP), and Songkhla (SK1, SK2, and SK3) provinces. Images obtained and modified from Google Earth Pro version 7.3.4.8248 (https://www.google.com/earth/about/).

Biting midges were collected in the vicinity of the residences of patients diagnosed with autochthonous leishmaniasis in five distinct geographical areas (Fig. 1). Insect samples were collected from three different locations in Songkhla Province where leishmaniasis cases were present, including sampling location 1 (SK1) (6°38′08.7″N, 100°25′35.9″E) and sampling location 2 (SK2) (6°36′14″N, 100°28′57″E) in Sadao District, and sampling location 3 (SK3) (6°44′30″N, 100°41′30″E) in Na Thawi District. The northern region was represented by two sampling locations: Meuang District, Chiang Rai Province (19°51′05.8″N, 99°39′37.1″E), and Wang Nuea District, Lampang Province (19°10′30″N, 99°38′51″E). Four Centers for Disease Control and Prevention (CDC) miniature light traps (25 W bulb) with ultraviolet (UV) light were placed at an approximate height of 1.5 m above the ground and operated from 18:00 to 6:00 h for two consecutive nights at each study site in July and August 2025. The collection bags were cooled on ice for 30 min to anesthetize the insects, then female midges were sorted from other insects by morphological characteristics under a stereomicroscope (Olympus, Tokyo, Japan). In the present study, all collected individuals were in the parous stage, and no gravid specimens were observed. Nulliparous individuals were excluded from the analysis because they have not yet taken a blood meal and are therefore unlikely to have been exposed to infection. Blood-engorged specimens were also excluded because, although parasite detection is feasible in these individuals, the presence of parasite DNA may reflect residual DNA derived from the host blood meal. Female biting midges were preserved in 1.5-ml microcentrifuge tubes containing 80% ethanol and subsequently transported to the Center of Excellence in Vector Biology and Vector-Borne Diseases, Department of Parasitology, Faculty of Medicine, Chulalongkorn University, for further investigation.

### 2.2 Non-destructive DNA extraction from *Culicoides* spp.

Before DNA extraction, sterilized distilled water was added to remove residual ethanol. Genomic DNA was extracted from individual parous female *Culicoides* samples using a non-destructive protocol with modifications. Briefly, 100 μl of cell lysis solution (GeneAll, Seoul, Korea) and 10 μl of proteinase K solution were added to each sample and incubated at 50 °C for 16 h. The lysate solution was used to extract DNA according to the instructions using Genti™32 Automated Nucleic Acid Extraction (GeneAll, Seoul, Korea). The concentration and quality of the DNA were measured using a NanoDrop 2000c spectrophotometer (Thermo Fisher Scientific, Waltham, USA). The whole *Culicoides* body was stored in a 1.5-ml microcentrifuge tube containing 80% ethanol solution for retrospective further morphological species identification.

### 2.3 Morphological and molecular identification of *Culicoides* spp.

Unmounted *Culicoides* specimens were initially identified based on their wing spot patterns. For samples that were difficult to identify, the head, wings, and genitalia with spermathecae were dissected and removed under a stereomicroscope using a sterile needle in a drop of 0.9% normal saline. The samples were mounted on glass slides using Hoyer’s medium. *Culicoides* spp. were morphologically identified according to the taxonomic keys by Wirth and Hubert (1989), and the morphology of *C. mahasarakhamense* was characterized as described by Pramual et al. (2021b). The identification of *Culicoides* spp. based on morphology was confirmed by sequencing of the mitochondrial cytochrome *c* oxidase subunit 1 (*cox*1) gene. PCR was performed on randomly selected samples of each representative species using primers C1-J-1718 and C1-N-2191 (Dallas et al., 2003), and the PCR amplification protocol was performed as described by Mathieu et al. (2020).

### 2.4 PCR identification of *Wolbachia* and *Cardinium* in field-caught *Culicoides* spp.

The presence of *Wolbachia* and *Cardinium* endosymbionts was assessed through PCR amplification of specific gene fragments. For *Wolbachia*, a fragment of the *wsp* (*Wolbachia* surface protein) gene was amplified using the forward primer wsp81F (5′-TGG TCC AAT AAG TGA TGA AGA AAC-3′) and the reverse primer wsp691R (5′-AAA AAT TAA ACG CTA CTC CA-3′) (Zhou et al., 1998). Detection of *Cardinium* was performed by amplifying a highly conserved region of the 16S rRNA gene with primers CAR-SP-F (5′-CGG CTT ATT AAG TCA GTT GTG AAA TCC TAG-3′) and CAR-SP-R (5′-TCC TTC CTC CCG CTT ACA CG-3′) (Nakamura et al., 2009). Each PCR reaction master mix was performed in a total volume of 25 μl containing 6 μl of genomic DNA (approximately 5–10 ng), 12.5 μl of Green Hot Start PCR Master Mix Direct-Load 2× (Biotechrabbit, Berlin, Germany), 1 μl of each forward and reverse primer (10 μM), and 4.5 μl of sterile deionized water. The thermal cycling conditions included an initial denaturation at 95 °C for 3 min, followed by 35 cycles of denaturation at 95 °C for 1 min, annealing at 55 °C for *wsp* and 57 °C for 16S rRNA for 1 min, and extension at 72 °C for 1 min, with a final extension step at 72 °C for 5 min. Plasmid DNA containing the specific sizes of the *wsp* and 16S rRNA genes served as positive controls, while genomic DNA from *Culicoides* specimens confirmed to be free of *Wolbachia* and *Cardinium*, along with sterile deionized water, were included as negative controls. The expected size of the *wsp* PCR product is approximately 593–644 bp, depending on the *Wolbachia* strain, while that of the *Cardinium* PCR product is approximately 595 bp.

### 2.5 Detection of *Leishmania* DNA in *Culicoides* spp.

The presence of *Leishmania* DNA was detected using a conventional PCR assay targeting the internal transcribed spacer 1 (ITS1) region of the ribosomal DNA cluster. Amplification was performed with the forward primer LeF (5′-TCC GCC CGA AAG TTC ACC GAT A-3′) and the reverse primer LeR (5′-CCA AGT CAT CCA TCG CGA CAC G-3′) (Spanakos et al., 2008). PCR reactions were carried out using the Green Hot Start PCR Master Mix Direct-Load 2× (Biotechrabbit, Berlin, Germany) according to the manufacturer’s instructions. The PCR cycling conditions followed the protocols previously described by Srisuton et al. (2019) and Sunantaraporn et al. (2021). DNA extracted from *Leishmania martiniquensis* culture (MHOM/TH/2012/CULE1) served as the positive control, while male *Culicoides* and sterilized deionized water were used as the negative control.

### 2.6 DNA cloning and sequencing

The PCR products for *Leishmania* spp., *Wolbachia*, and *Cardinium* were inserted into the pGEM-T Easy Vector (Promega, Madison, WI, USA) using a rapid DNA ligation kit (Promega, Madison, WI, USA) following the manufacturer’s instructions. The plasmid DNA was transformed into *Escherichia coli* (DH5α) competent cells, and the transformants were screened by blue-white colony selection using a colony PCR assay. Three to five positive clones, presumed to contain the desired genes, were cultured in Luria-Bertani (LB) broth, with the addition of ampicillin (100 mg/ml). The chimeric DNA was extracted using the GeneAll® Exprep™ plasmid purification kit (GeneAll, Seoul, Korea) following the manufacturer’s instructions. The extracted DNA was then sent to a commercial service at Macrogen Inc., South Korea, for Sanger DNA sequencing.

### 2.7 Phylogenetic tree construction and haplotype network

All nucleotide sequences were subjected to manual editing and trimming prior to alignment using the Clustal W multiple alignment function in BioEdit sequence alignment editor v.7.2.5 (Hall, 1999). The consensus nucleotide sequences were compared to previously published sequences in the GenBank database using the Basic Local Alignment Search Tool (BLAST) online in the NCBI database (https://blast.ncbi.nlm.nih.gov/Blast.cgi). The phylogenetic relationships were reconstructed using the maximum likelihood (ML) method, based on the model with the lowest Bayesian Information Criterion (BIC) scores, as implemented in MEGA 11 (Tamura et al., 2021). The reliability of the phylogenetic tree was estimated based on 1000 bootstrap pseudoreplicates. The *Wolbachia* putative strain prediction was evaluated based on the analysis of the *wsp* sequences using the DnaSP software version 6 (Rozas et al., 2017). A Templeton-Crandall-Sing (TCS) network was constructed using PopART v.1.7 (Leigh and Bryant, 2015).

### 2.8 Statistical analysis

Species richness (the number of species in the study areas) and species relative abundance (the number of samples per species/total number of samples × 100) for each site were calculated. Prevalence was calculated as the proportion of PCR-positive samples relative to the total number of individuals examined for each *Culicoides* species, study site, and infection status (single infection and co-infection). Prevalences are presented as percentages with corresponding 95% confidence intervals (95% CI). The associations between the detection of *Leishmania* DNA and locations, *Culicoides* species, and endosymbiont presence were assessed using binary logistic regression. A *P*-value of < 0.05 was defined as statistically significant. All statistical analyses were performed using SPSS v.29 (IBM Corp., Armonk, NY, USA).

## 3 Results

### 3.1 Species composition of *Culicoides* biting midges

A total of 593 specimens of *Culicoides*, representing 21 species of *Culicoides* and one species of *Culicoides* (*Trithecoides*), were collected from five collection sites across Songkhla (SK1, SK2, and SK3) in the southern region and Chiang Rai and Lampang in the northern region of Thailand. Species richness of *Culicoides* spp. demonstrated the lowest richness observed in SK1 (5 species) and the highest in Lampang (16 species). Overall species richness across all sampling locations was 21 species. The relative abundance of *Culicoides* spp. differed across sampling locations. Lampang contributed the high relative abundance of *Culicoides* samples (36.76%; 218/593), followed by Chiang Rai (26.98%; 160/593). In southern Thailand, SK2 showed the highest relative abundance (17.87%; 106/593), followed by SK3 (13.49%; 80/593) and SK1 (4.89%; 29/593). *Culicoides peregrinus* was the most frequent species (*n* = 127), accounting for 21.4% of all collected samples, followed by *C. guttifer* (*n* = 78), *C. orientalis* (*n* = 59), *C. oxystoma* (*n* = 48), and *C. shortti* (*n* = 48). The *Culicoides* species composition varied among sampling sites, with distinct dominant species observed at each location. In SK1, *C. oxystoma* was the most abundant species (*n* = 11; 37.9%). For SK2, *C. peregrinus* predominated (*n* = 85; 80.2%), whereas SK3 was dominated by *C. guttifer* (*n* = 49; 61.3%). *Culicoides tainanus* (*n* = 35; 21.9%) and *C. orientalis* (*n* = 34; 21.3%) were mainly found in Chiang Rai. In addition, *C. shortti* (*n* = 42; 19.3%) was the most frequently collected species in Lampang (Table 1 and Fig. 1).

**Table 1:** Species of *Culicoides* collected from five locations with leishmaniasis cases in three provinces of Thailand.

Species	Study site					Total
	Songkhla			Chiang Rai	Lampang	
	SK1	SK2	SK3			
*C. actoni*	0	0	0	0	3	3
*C. arakawae*	0	0	1	0	0	1
*C. fulvus*	0	0	0	0	4	4
*C. guttifer*	0	3	49	0	26	78
*C. huffi*	2	0	4	8	2	16
*C. innoxius*	0	0	3	1	11	15
*C. insignipennis*	0	1	0	10	5	16
*C. jacobsoni*	0	0	0	31	2	33
*C. liui*	0	0	0	7	0	7
*C. mahasarakhamense*	3	7	7	0	25	42
*C. malayae*	0	0	0	4	0	4
*C. orientalis*	1	3	2	34	19	59
*C. oxystoma*	11	7	0	6	24	48
*C. palpifer*	0	0	7	0	9	16
*C. parahumeralis*	0	0	2	0	1	3
*C. peregrinus*	12	85	4	0	26	127
*C. shortti*	0	0	0	6	42	48
*C. sumatrae*	0	0	0	1	11	12
*C. tainanus*	0	0	0	35	0	35
*C. tamada*	0	0	1	0	0	1
*C. tenuipalpis*	0	0	0	0	3	3
*C.* (*Trithecoides*) sp.	0	0	0	17	5	22
Total	29	106	80	160	218	593
Relative abundance	4.89	17.87	13.49	26.98	36.76	
Species richness	5	6	9	10	16	21

*Note*: The calculation of species richness was based on all identified species, except for *C*. (*Trithecoides*) sp.

*Abbreviations*: SK1 and SK2, Sadao District; SK3, Na Thawi District.

### 3.2 Detection of *Wolbachia* and *Cardinium* endosymbionts in *Culicoides* spp.

Conventional PCR was used to amplify the *Wolbachia* surface protein (*wsp*) gene in several *Culicoides* spp. Overall, a single *Wolbachia* infection was detected in 18 *Culicoides* species, with a prevalence of 29.01% (95% CI: 25.49–32.79%). In Songkhla, the prevalence exhibited variation among the collection sites, with 34.48% (95% CI: 19.85–52.74%), 21.70% (95% CI: 14.86–30.52%), and 12.50% (95% CI: 6.74–21.69%) recorded at SK1, SK2, and SK3, respectively. In northern Thailand, the prevalence of *Wolbachia* infection was 31.88% (95% CI: 25.14–39.46%) in Chiang Rai and 35.78% (95% CI: 29.71–42.34%) in Lampang.

The presence of *Cardinium* infection was detected through the amplification of the 16S rRNA gene. Among the 593 specimens examined, 86 (14.50%, 95% CI: 11.89–17.57%) were found to be positive for *Cardinium*. In Songkhla, the prevalences were 3.45% (95% CI: 0–18.63%), 10.38% (95% CI: 5.74–17.79%), and 48.75% (95% CI: 38.11–59.51%) at sampling locations SK1, SK2, and SK3, respectively. In northern Thailand, the prevalence of *Cardinium* infection was 1.25% (95% CI: 0–4.73%) in Chiang Rai and 15.14% (95% CI: 10.95–20.53%) in Lampang. *Cardinium* infection was most frequently detected in *C. guttifer* and *C. mahasarakhamense*.

Furthermore, co-infection with *Wolbachia* and *Cardinium* endosymbionts was observed in several *Culicoides* species, with an overall prevalence of 11.47% (95% CI: 9.14–14.30%). Co-infections were identified at three collection sites: SK3 in Songkhla (13.75%, 95% CI: 7.68–23.14%), Chiang Rai (7.50%, 95% CI: 4.23–12.77%), and Lampang (20.64%, 95% CI: 15.78–26.52%) (Table 2).

**Table 2:** Detection of endosymbionts and *Leishmania* parasite in *Culicoides* spp. from northern and southern Thailand.

Location/*Culicoides* spp.	*N*	Molecular detection of endosymbionts			*Leishmania* detection	Prevalence (95% CI) (%)			
		Single infection		Co-infection		Single infection		Co-infection	*Leishmania*
		W	C	W + C		W	C	W + C	
**Songkhla (SK1)**									
*C. huffi*	2	0	0	0	0	0	0	0	0
*C. mahasarakhamense*	3	1	1	0	0	33.33 (5.63–79.76)	33.33 (5.63–79.76)	0	0
*C. orientalis*	1	0	0	0	0	0	0	0	0
*C. oxystoma*	11	6	0	0	0	54.55 (27.99–78.75)	0	0	0
*C. peregrinus*	12	3	0	0	0	25.00 (8.27–53.85)	0	0	0
Subtotal	29	10	1	0	0	34.48 (19.85–52.74)	3.45 (0–18.63)	0	0
**Songkhla (SK2)**									
*C. guttifer*	3	0	3	0	0	0	100 (38.25–100)	0	0
*C. insignipennis*	1	0	0	0	0	0	0	0	0
*C. mahasarakhamense*	7	0	6	0	0	0	85.71 (46.65–99.47)	0	0
*C. orientalis*	3	0	0	0	0	0	0	0	0
*C. oxystoma*	7	1	0	0	2	14.29 (0.53–53.35)	0	0	28.57 (7.56–64.76)
*C. peregrinus*	85	22	2	0	7	25.88 (17.70–36.15)	2.35 (0.14–8.68)	0	8.24 (3.79–16.29)
Subtotal	106	23	11	0	9	21.70 (14.86–30.52)	10.38 (5.74–17.79)	0	8.49 (4.35–15.54)
**Songkhla (SK3)**									
*C. arakawae*	1	0	0	0	0	0	0	0	0
*C. guttifer*	49	1	37	8	3	2.04 (0–11.69)	75.51 (61.78–85.53)	16.33 (8.25–29.30)	6.12 (1.48–17.15)
*C. huffi*	4	1	0	0	0	25.00 (3.41–71.09)	0	0	0
*C. innoxius*	3	0	0	0	0	0	0	0	0
*C. mahasarakhamense*	7	0	2	3	2	0	28.57 (7.56–64.76)	42.86 (15.75–75.02)	28.57 (7.56–64.76)
*C. orientalis*	2	1	0	0	0	50.00 (9.45–90.55)	0	0	0
*C. parahumeralis*	2	1	0	0	0	50.00 (9.45–90.55)	0	0	0
*C. peregrinus*	4	3	0	0	0	75.00 (28.91–96.59)	0	0	0
*C. tamada*	1	1	0	0	0	100 (16.75–100)	0	0	0
*C. palpifer*	7	2	0	0	0	28.57 (7.56–64.76)	0	0	0
Subtotal	80	10	39	11	5	12.50 (6.74–21.69)	48.75 (38.11–59.51)	13.75 (7.68–23.14)	6.25 (2.36–14.15)
Total (Songkhla)	215	43	51	11	14	20.00 (15.18–25.88)	23.72 (18.51–29.86)	5.12 (2.78–9.03)	6.51 (3.83–10.72)
**Chiang Rai**									
*C. jacobsoni*	31	9	0	1	3	29.03 (15.94–46.75)	0	3.23 (0–17.58)	9.68 (2.56–25.69)
*C. orientalis*	34	11	0	2	3	32.35 (19.04–49.25)	0	5.88 (0.65–20.07)	8.82 (2.29–23.72)
*C. tainanus*	35	11	0	2	3	31.43 (18.45–48.08)	0	5.71 (0.62–19.57)	8.57 (2.21–23.13)
*C. huffi*	8	5	1	0	1	62.50 (30.38–86.51)	12.50 (0.11–49.22)	0	12.50 (0.11–49.22)
*C. innoxius*	1	0	0	0	0	0	0	0	0
*C. insignipennis*	10	5	0	0	2	50.00 (23.66–76.34)	0	0	20.00 (4.59–52.06)
*C. liui*	7	3	0	4	2	42.86 (15.75–75.02)	0	57.14 (24.98–84.25)	28.57 (7.56–64.76)
*C. malayae*	4	0	0	3	0	0	0	75.00 (28.91–96.59)	0
*C. sumatrae*	1	0	0	0	0	0	0	0	0
*C. oxystoma*	6	2	0	0	0	33.33 (9.25–70.43)	0	0	0
*C. shortti*	6	0	0	0	0	0	0	0	0
*C.* (*Trithecoides*) sp.	17	5	1	0	1	29.41 (12.99–53.43)	5.88 (0–28.92)	0	5.88 (0–28.92)
Total	160	51	2	12	15	31.88 (25.14–39.46)	1.25 (0–4.73)	7.50 (4.23–12.77)	9.38 (5.67–14.99)
**Lampang**									
*C. actoni*	3	1	0	1	1	33.33 (5.63–79.76)	0	33.33 (5.63–79.76)	33.33 (5.63–79.76)
*C. fulvus*	4	0	1	1	1	0	25.00 (3.41–71.09)	25.00 (3.41–71.09)	25.00 (3.41–71.09)
*C. guttifer*	26	0	16	10	3	0	61.54 (42.48–77.63)	38.46 (22.37–57.52)	11.54 (3.18–29.80)
*C. huffi*	2	1	0	0	0	50.00 (9.45–90.55)	0	0	0
*C. innoxius*	11	5	0	2	1	45.45 (21.25–72.01)	0	18.18 (3.99–49.85)	9.09 (0–39.91)
*C. insignipennis*	5	4	0	0	1	80.00 (35.96–97.97)	0	0	20.00 (2.03–64.04)
*C. jacobsoni*	2	2	0	0	0	100 (29.02–100)	0	0	0
*C. mahasarakhamense*	25	2	9	12	5	8.00 (1.09–26.10)	36.00 (20.16–55.57)	48.00 (30.03–66.50)	20.00 (8.41–39.58)
*C. orientalis*	19	9	0	6	2	47.37 (27.33–68.30)	0	31.58 (15.16–54.20)	10.53 (1.70–32.63)
*C. oxystoma*	24	12	2	2	1	50.00 (31.43–68.57)	8.33 (1.16–27.00)	8.33 (1.16–27.00)	4.17 (0–21.87)
*C. peregrinus*	26	11	2	3	3	42.31 (25.52–61.08)	7.69 (1.02–25.26)	11.54 (3.18–29.80)	11.54 (3.18–29.80)
*C. shortti*	42	24	0	4	5	57.14 (42.19–70.89)	0	9.52 (3.21–22.62)	11.90 (4.73–25.46)
*C. parahumeralis*	1	0	0	0	0	0	0	0	0
*C. sumatrae*	11	3	0	1	1	27.27 (9.20–57.11)	0	9.09 (0–39.91)	9.09 (0–39.91)
*C. tenuipalpis*	3	0	0	1	0	0	0	33.33 (5.63–79.76)	0
*C. palpifer*	9	1	3	1	0	11.11 (0.00–45.67)	33.33 (11.73–64.91)	11.11 (0–45.67)	0
*C.* (*Trithecoides*) sp.	5	3	0	1	0	60.00 (22.91–88.40)	0	20.00 (2.03–64.04)	0
Total	218	78	33	45	24	35.78 (29.71–42.34)	15.14 (10.95–20.53)	20.64 (15.78–26.52)	11.01 (7.46–15.91)
Grand total	593	172	86	68	53	29.01 (25.49–32.79)	14.50 (11.89–17.57)	11.47 (9.14–14.30)	8.94 (6.88–11.52)

*Abbreviations*: *N*, no. of tested; SK1 and SK2, Sadao district; SK3, Na Thawi district; W, *Wolbachia*, C, *Cardinium*.

### 3.3 Phylogenetic relationships and *Wolbachia* putative strain identification

**Fig. 2:**
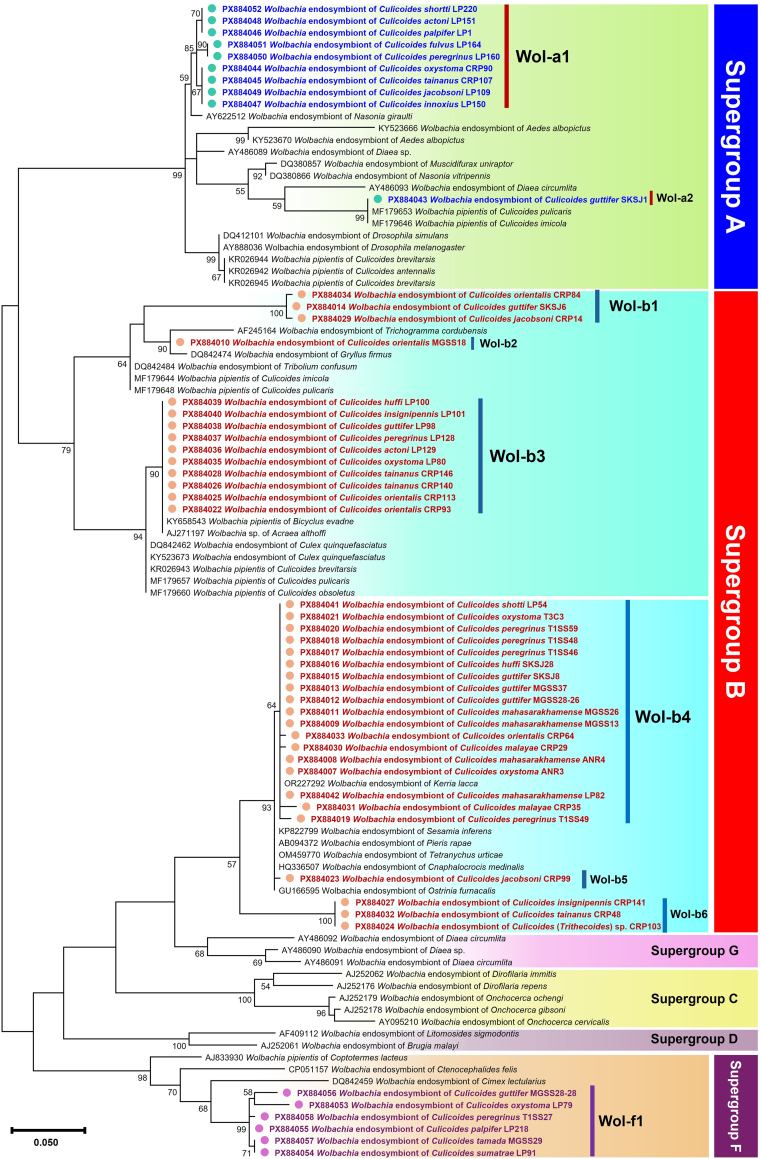
Phylogenetic relationships among *Wolbachia* supergroups inferred from partial *wsp* sequences. The maximum likelihood phylogenetic tree was generated using the Kimura 2-parameter model with gamma distribution (K2+G) and includes representative *Wolbachia* reference sequences retrieved from the GenBank database.

Partial *wsp* sequences were successfully obtained from 52 *Wolbachia*-infected *Culicoides* samples, representing all morphologically identified species found to harbor *Wolbachia* endosymbionts. BLAST analysis revealed that all *wsp* sequences were identical to those of *Wolbachia* bacterial endosymbionts, with sequence similarities ranging from 91.43% to 100% (Supplementary Table S1). Phylogenetic analysis of these *Wolbachia*-positive sequences revealed their allocation to three major *Wolbachia* supergroups: Supergroup A (*n* = 10), Supergroup B (*n* = 36), and Supergroup F (*n* = 6). In one sample from *C. guttifer*, a co-infection was observed with *Wolbachia* Supergroup B (isolate MGSS28-26) and Supergroup F (isolate MGSS28-28). According to phylogenetic analysis, the majority of *wsp* sequences were assigned to *Wolbachia* Supergroup B and clustered into six putative strains: Wol-b1, Wol-b2, Wol-b3, Wol-b4, Wol-b5, and Wol-b6. Two putative strains, Wol-a1 and Wol-a2, belonged to *Wolbachia* Supergroup A, and one putative strain, Wol-f1, was classified within Supergroup F (Fig. 2).

**Fig. 3:**
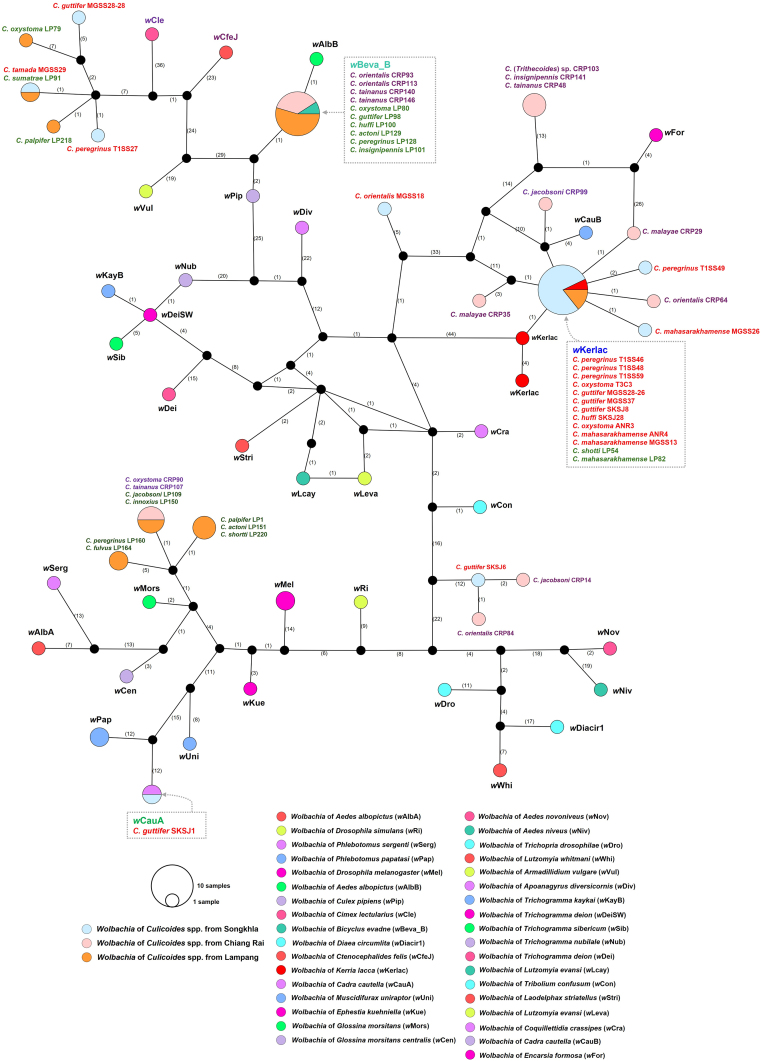
Genetic diversity and relationships among putative *Wolbachia* strains inferred from partial *wsp* sequences using a TCS haplotype network.

Haplotype network analysis of the newly characterised *Wolbachia* strains and 35 strains previously reported by Zhou et al. (1998) demonstrated that the *wsp* sequences clustered into three *Wolbachia* putative strains, which is consistent with previous classifications. The first cluster corresponded to the *w*Kerlac strain (*Wolbachia* of *Kerria lacca*) from Wol-b4 and comprised endosymbionts from 10 *Culicoides* samples: *C. peregrinus* (*n* = 3), *C. guttifer* (*n* = 3), *C. mahasarakhamense* (*n* = 2), *C. oxystoma* (*n* = 1), and *C. huffi* (*n* = 1) collected from Songkhla, and one each, *C. shortti* (LP54) and *C. mahasarakhamense* (LP82), from Lampang. *Wolbachia* from several additional *Culicoides* species exhibited close genetic similarity to *w*Kerlac, differing by only 1–2 base pairs (Fig. 3). These species included *C. malayae* (CRP29) and *C. orientalis* (CRP64) from Chiang Rai, and *C. mahasarakhamense* (MGSS26) and *C. peregrinus* (T1SS49) from Songkhla. The second cluster, corresponding to the *w*Beva_B strain (*Wolbachia* of *Bicyclus evadne*) from Wol-b3, was identified in *C. orientalis* (*n* = 2) and *C. tainanus* (*n* = 2) from Chiang Rai, and in six *Culicoides* spp. including *C. oxystoma*, *C. guttifer*, *C. huffi*, *C. actoni*, *C. peregrinus*, and *C. insignipennis* collected from Lampang. The third cluster, *w*CauA strain (*Wolbachia* of *Cadra cautella*) from Wol-a2, was identified in *C. guttifer* from Songkhla. Other *Wolbachia* variants detected in this study exhibited close genetic relationships to strains previously reported from a range of different insect hosts (Fig. 3).

### 3.4 Phylogenetic relationships of *Cardinium* endosymbionts

A total of 52 *Cardinium*-positive samples from 16 *Culicoides* spp. were randomly selected for 16S rRNA sequencing. BLAST analysis of these sequences against the GenBank database revealed 97.98–99.83% similarity with “*Candidatus* Cardinium hertigii” (GenBank: KR026922) previously reported in *Culicoides williwilli*. Additionally, eleven 16S rRNA sequences from multiple *Culicoides* species showed 98.65–98.99% similarity with the *Cardinium* endosymbiont of *Microzetorchestes emeryi* (GenBank: MG889459) (Supplementary Table S2).

**Fig. 4:**
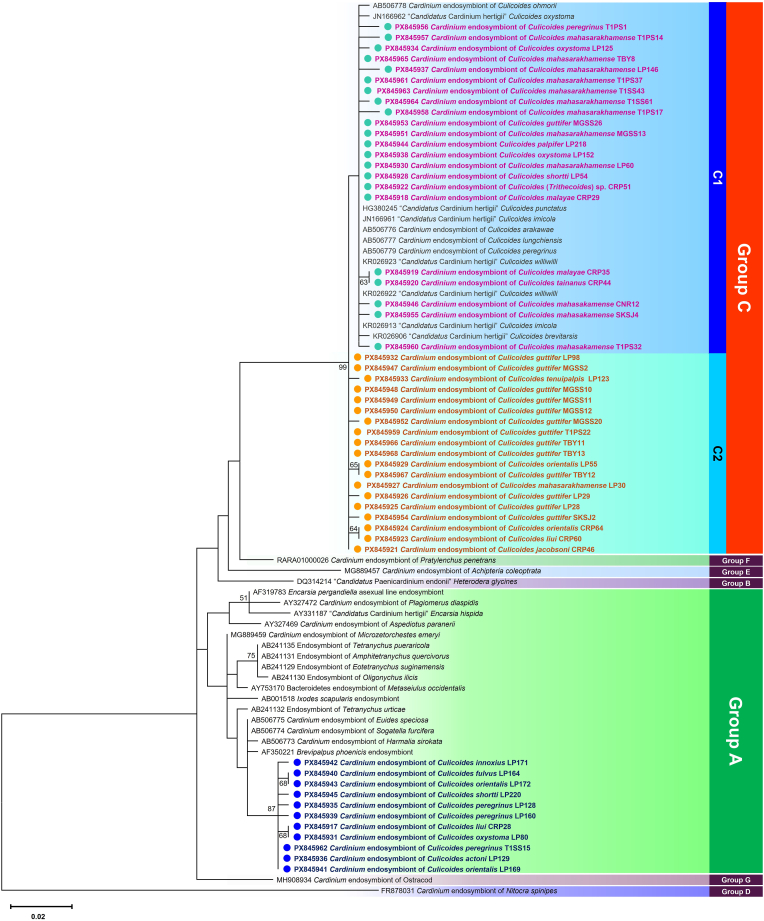
Phylogenetic relationship of *Cardinium* endosymbionts based on partial 16S rRNA sequences. The maximum likelihood tree was constructed using the Kimura 2-parameter model with gamma distribution (K2+G) and includes *Cardinium* sequences from *Culicoides* spp. obtained in this study and reference sequences from the GenBank database.

The phylogenetic analysis classified the *Cardinium* sequences into two major groups, designated as Group A and Group C. The majority of sequences (*n* = 41) clustered within *Cardinium* Group C, which was further divided into two subgroups (Fig. 4). Subgroup C1 comprised 22 *Cardinium* sequences detected in 9 *Culicoides* spp., including reference sequences from *Cardinium* previously reported in various *Culicoides* species. Subgroup C2 contained 19 sequences identified in six *Culicoides* spp. from the present study (Fig. 4). Notably, eleven *Cardinium*-positive samples were phylogenetically classified for the first time within *Cardinium* Group A (Fig. 4).

### 3.5 Detection of *Leishmania* DNA in *Culicoides* spp.

A total of 593 extracted DNA samples were subjected to molecular analysis in order to determine the presence of *Leishmania* DNA in *Culicoides* spp. samples. The results demonstrated the presence of *Leishmania* DNA in 53 (8.94%, 95% CI: 6.88–11.52%) *Culicoides* samples collected from Chiang Rai (*n* = 15, 9.38%, 95% CI: 5.67–14.99%), Lampang (*n* = 24, 11.01%, 95% CI: 7.46–15.91%), and Songkhla (*n* = 14, 6.51%, 95% CI: 3.83–10.72%). No *Leishmania* DNA was detected at the SK1 sampling site in Songkhla (Table 2).

The BLAST analysis of positive *Leishmania* samples based on ITS1 sequences revealed that a single infection of *L. martiniquensis* occurred in 45 samples from all sampling sites. In contrast, a single infection with *L. orientalis* was identified in two *C. orientalis* samples collected in Lampang. Co-infection of *L. martiniquensis* + *L. orientalis* was demonstrated in six samples from two sampling sites from Lampang (four samples of *C. guttifer*, *C. shortti*, *C. peregrinus*, and *C. fulvus*) and Chiang Rai (two samples of *C. jacobsoni* and *C. tainanus*) (Supplementary Table S3).

### 3.6 Co-occurrence of *Leishmania* parasites and endosymbionts in *Culicoides* spp.

The binary logistic regression analysis showed that neither sampling location nor *Culicoides* species was significantly associated with *Leishmania* detection in either crude or adjusted models. Although higher crude odds were observed in Chiang Rai (OR = 1.49, 95% CI: 0.69–3.17) and Lampang (OR = 1.78, 95% CI: 0.89–3.53) compared with Songkhla, these associations were not significant after adjustment. No significant associations were also detected between *Leishmania* detection and individual *Culicoides* species. While *Culicoides mahasarakhamense* showed a higher crude odds ratio (OR = 2.20, 95% CI: 0.60–8.12), this was not retained in the adjusted model (adjusted OR = 0.82, 95% CI: 0.19–3.62, *P* = 0.799).

**Fig. 5:**
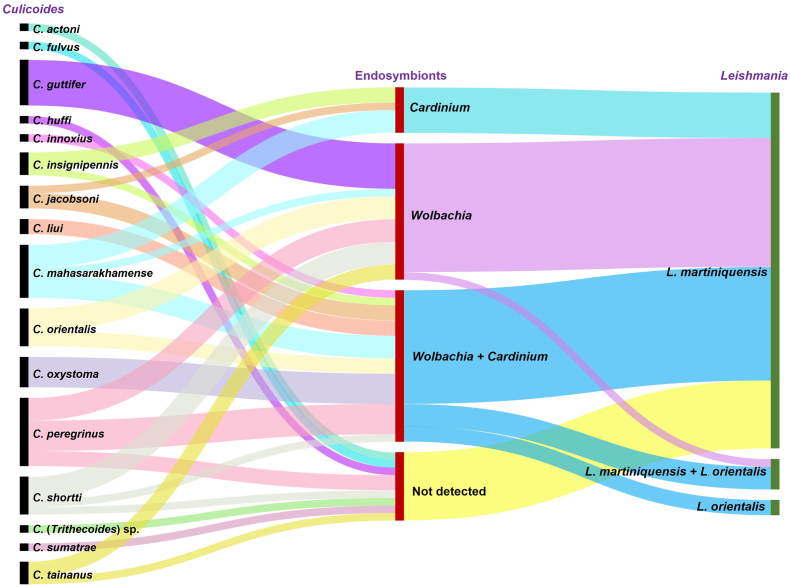
An alluvial diagram illustrating the associations between *Culicoides* species, bacterial endosymbionts, and *Leishmania* spp.

In contrast, endosymbiont infections were significantly associated with *Leishmania* detection after adjustment. *Wolbachia*-positive samples had higher odds of *Leishmania* detection than negative samples (adjusted OR = 3.51, 95% CI: 1.52–8.12, *P* = 0.003). Similarly, *Cardinium* positivity was associated with increased odds (adjusted OR = 4.21, 95% CI: 1.15–15.38; *P* = 0.030), despite a non-significant crude association. Co-infection with *Wolbachia* and *Cardinium* showed a strong association, with markedly higher odds of *Leishmania* detection compared with non-co-infected samples (adjusted OR = 17.70, 95% CI: 6.53–48.03, *P* < 0.001) (Table 3). A tripartite network analysis demonstrating the associations among *Culicoides* species, bacterial endosymbionts, and *Leishmania* spp. detection is presented in Fig. 5. The data on positive *Leishmania* DNA and associated endosymbionts, including both single infections and co-infections of *Wolbachia* and *Cardinium*, are presented in Supplementary Table S3.

**Table 3:** Association between *Leishmania* detection and sampling location, species of *Culicoides*, and endosymbiont infections.

Variable	*Leishmania*-positive (*n*, %)	*Leishmania*-negative (*n*, %)	Crude OR (95% CI)	Adjusted OR (95% CI)	*P*-value
**Location**					
Songkhla	14 (6.5)	201 (93.5)	1 (reference)		
Chiang Rai	15 (9.4)	145 (90.6)	1.49 (0.69–3.17)	1.56 (0.50–4.89)	0.448
Lampang	24 (11.0)	194 (89.0)	1.78 (0.89–3.53)	0.90 (0.37–2.14)	0.806
***Culicoides* species**^a^					
*C. oxystoma*	4 (8.3)	44 (91.7)	1 (reference)		
*C. orientalis*	5 (8.5)	54 (91.5)	1.02 (0.26–4.02)	0.63 (0.14–2.83)	0.550
*C. shortti*	5 (10.4)	43 (89.6)	1.28 (0.32–5.09)	1.14 (0.26–4.89)	0.862
*C. guttifer*	6 (7.7)	72 (92.3)	0.92 (0.24–3.43)	0.37 (0.08–1.74)	0.208
*C. mahasarakhamense*	7 (16.7)	35 (83.3)	2.20 (0.60–8.12)	0.82 (0.19–3.62)	0.799
*C. peregrinus*	9 (7.1)	118 (92.9)	0.84 (0.25–2.86)	1.09 (0.29–4.07)	0.894
Other species	17 (8.9)	174 (91.1)	1.07 (0.34–3.35)	0.74 (0.20–2.68)	0.645
***Wolbachia***					
Negative	35 (8.3)	386 (91.7)	1 (reference)		
Positive	18 (10.5)	154 (89.5)	1.29 (0.71–2.34)	3.51 (1.52–8.12)	0.003∗∗
***Cardinium***					
Negative	47 (9.3)	460 (90.7)	1 (reference)		
Positive	6 (7.0)	80 (93.0)	0.73 (0.30–1.77)	4.21 (1.15–15.38)	0.03∗
***Wolbachia* + *Cardinium* co-infection**					
Negative	33 (6.3)	492 (93.7)	1 (reference)		
Positive	20 (29.4)	48 (70.6)	6.21 (3.31–11.66)	17.70 (6.53–48.03)	<0.001∗∗∗

*Note*: ∗*P* < 0.05; ∗∗*P* < 0.01; ∗∗∗*P* < 0.001.

a Six *Culicoides* species were included in the analysis for individual comparisons.

## 4 Discussion

The present study investigates the prevalence of two bacterial endosymbionts, *Wolbachia* and *Cardinium*, in *Culicoides* biting midges collected from affected areas of autochthonous leishmaniasis in northern and southern Thailand. The overall *Wolbachia* infection rate was 29.0%, indicating a moderately high prevalence in natural populations. This finding contrasts with previous reports showing lower *Wolbachia* prevalence rates in *Culicoides* species collected in Spain (6.2%) (Pagès et al., 2017) and Australia (6.4%) (Mee et al., 2015). The prevalences of *Wolbachia* detected in this study are closely aligned with those previously observed in the USA (22.0%) (Covey et al., 2020). *Wolbachia* infection was detected in multiple *Culicoides* species, including *C. mahasarakamense*, *C. peregrinus*, *C. oxystoma*, *C. guttifer*, *C. jacosoni*, and *C. orientalis*. These species have also been previously identified as *Leishmania*-positive in areas with a documented history of leishmaniasis, as reported by Sunantaraporn et al. (2021), Songumpai et al. (2022), Ampol et al. (2024), and Promrangsee et al. (2024). As demonstrated in a previous report by Pagès et al. (2017), *Wolbachia* has been identified in *C. imicola*, *C. obsoletus* (*s.l*.), and *C. pulicaris* (*s.l*.), which are considered Palaearctic vectors of bluetongue and Schmallenberg diseases. Low-level *Wolbachia* infection was identified in 10 *Culicoides* species from Australia (Mee et al., 2015). The *Wolbachia* infection was identified in *C. crepuscularis*, *C. debilipalpis*, *C. edeni*, *C. haematopotus*, *C. insignis*, *C*. *venustus*, and *C. sonorensis* from the USA (Covey et al., 2020).

The 16S rRNA, *ftsZ*, and *wsp* genes have been frequently employed as genetic markers for the classification of the eight supergroups (A to H) of *Wolbachia* (Augustinos et al., 2011; Wang et al., 2014; Glowska et al., 2015). The most common *Wolbachia* supergroups in arthropods are A and B (Breeuwer et al., 1992; Werren et al., 1995). Phylogenetic analysis revealed that the *Wolbachia* isolates detected in *Culicoides* spp. in the present study were genetically clustered into supergroups A, B, and F. The majority of our samples were phylogenetically separated into multiple clades within Supergroup B. *Wolbachia* sequences previously detected in Australian and Spanish *Culicoides* spp. were segregated into two distinct clades within Supergroup B (Mee et al., 2015; Pagès et al., 2017). The *Wolbachia* Supergroup F has been identified in filarial nematodes, cat fleas, termites, and bed bugs (Driscoll et al., 2020; Zimmermann et al., 2021; Chebbah et al., 2023; Sinha et al., 2023). It was a notable finding of this study that some *Wolbachia* isolates from *Culicoides* spp. were detected in the Supergroup F. It is noteworthy that co-infection with supergroups B and F was observed in *C. guttifer* in Songkhla.

The overall infection rate of *Cardinium* was 14.5%, detected across multiple *Culicoides* species, indicating a moderate prevalence in this study. Previous investigations have reported varying prevalences of *Cardinium* infection in different regions, including Australia (25.5%), Japan (16.0%), and Spain (1.1%) (Nakamura et al., 2009; Mee et al., 2015; Pagès et al., 2017). Previously, several reports have identified the *Cardinium* endosymbiont in relevant Palaearctic *Culicoides* species: *C. imicola*, *C. pulicaris*, and *C. punctatus*. A study by Pagès et al. (2017) has demonstrated the presence of *Cardinium* infection in a wide range of *C. obsoletus* (*s.l*.), *C. festivipennis*, *C. flavipulicaris*, *C. haranti*, *C. maritimus*, *C. minutissimus*, *C*. *newsteadi*, *C. punctatus*, and *C. sahariensis*. *Cardinium* infections were observed in several vector species, including *C. imicola* and the Pulicaris species complex (*C. pulicaris*, *C. bysta*, *C. newsteadi*, and *C. punctatus*) (Pilgrim et al., 2021). The most prevalent *Culicoides* species infected with *Cardinium* was *C. guttifer* (94.87%; 74/78), followed by *C. mahasarakhamense* (78.57%; 33/42). High *Cardinium* prevalence in *Culicoides* species is likely influenced by several factors. First, species-specific variation plays an important role, as previous studies have shown that certain species, such as *C*. *imicola* and members of the *C. pulicaris* complex, often exhibit moderate to high infection rates, whereas others, including the *C*. *obsoletus* species group, may display low or undetectable levels of infection (Pilgrim et al., 2021). Three *Culicoides* species (*C. arakawae*, *C. ohmorii*, and *C. peregrinus*) exhibited a 100% *Cardinium* infection rate, as all examined individuals tested positive (Nakamura et al., 2009). Secondly, environmental factors have been implicated, with evidence suggesting that conditions such as land surface temperature are strongly associated with infection prevalence. In particular, higher temperatures during early developmental stages may promote increased infection frequencies in adult midges (Morag et al., 2012, 2013). Thirdly, habitat-related differences may also contribute to variation in prevalence. For instance, *Culicoides* populations collected near livestock premises have been reported to exhibit significantly higher *Cardinium* infection rates compared to those from more natural habitats, indicating that host-associated ecological factors may influence the distribution and maintenance of this endosymbiont (Pagès et al., 2017). In this study, the observed *Cardinium* prevalence may be shaped by a combination of habitat-related and species-specific factors. Notably, *C. guttifer* and *C. mahasarakhamense* were both collected from livestock-associated environments, specifically chicken coops (Jomkumsing et al., 2021; Sunantaraporn et al., 2022; Tepboonrueng et al., 2026), which may promote higher infection frequencies due to shared ecological conditions and increased opportunities for symbiont persistence and transmission. Accordingly, these species represent the most suitable candidates for examining the influence of *Cardinium* on vectorial capacity (Pilgrim et al., 2021). The present study demonstrates the occurrence of co-infections with *Wolbachia* and *Cardinium* in several *Culicoides* species, with a moderate prevalence of 11.47%. Previous studies have reported substantially higher co-infection rates in other insect groups, such as planthoppers, where a prevalence of 32.7% has been observed (Nakamura et al., 2009). However, to date, co-infection of these endosymbionts has not been reported in *Culicoides* biting midges. A phylogenetic analysis of *Cardinium* based on 16S rRNA sequences revealed the existence of seven distinct groups: A, B, C, D, E, F, and G (Tarlachkov et al., 2023). In the present study, the majority of *Cardinium*-positive samples were grouped in Group C, which contained *Cardinium* endosymbionts detected in diverse *Culicoides* species from regions worldwide. Furthermore, the 16S rRNA sequences of the *Cardinium* strains were genetically grouped into two subgroups within Group C (C1 and C2). Furthermore, this is the first report of *Cardinium* being classified into Group A in *Culicoides* spp. A previous report has indicated that *Cardinium* Group A has been identified in several insect groups, including hemipterans, hymenopterans, opilionids, mites, and spiders (Noel and Atibalentja, 2006).

This study identified *Wolbachia* putative strains using a TCS haplotype network, which illustrates the genealogical relationships among strains detected in host populations. Genetic variation (mutations) in *Wolbachia* surface protein (*wsp*) gene was analyzed to determine the relatedness among strains, infer ancestral lineages, and assess their distribution across different host species. The *Wolbachia* endosymbiont networks in *Culicoides* biting midges were grouped according to the *Wolbachia* putative strains previously described by Zhou et al. (1998) based on *wsp* sequences. In this study, three *Wolbachia* putative strains were identified as *w*Kerlac (*Wolbachia* of *Kerria lacca*), *w*Beva_B (*Wolbachia* of *Bicyclus evadne*), and *w*CauA (*Wolbachia* of *Cadra cautella*). The effects of *Wolbachia* infection are diverse and include inhibiting arbovirus, malaria, and filarial nematode development, as well as inducing various reproductive abnormalities (Stouthamer et al., 1999). The results of this study suggest that the putative *Wolbachia* strains identified may play a role in determining sexual characteristics and the sex ratio of insect populations (Sasaki et al., 2005; Vashishtha et al., 2011; Duplouy and Brattström, 2018). In the present study, *Wolbachia* infection was classified solely based on the *wsp* gene. The separation of the clades into distinct *Wolbachia* Supergroup B sequences indicated a high degree of divergence, consistent with the expectation that the strain would be novel. To accurately characterize the novel *Wolbachia* strain, a multi-locus sequence typing (MLST) analysis should be conducted using the *gatB*, *coxA*, *hcpA*, *fbpA*, and *ftsZ* genes (Baldo et al., 2006). This approach will facilitate a more precise delineation of the *Wolbachia* strains.

Detection of *Leishmania* parasites was carried out using an ITS1-PCR assay, revealing an overall prevalence of 8.94%. Although *Leishmania* DNA was identified using this molecular approach, these results do not confirm vector competence. A key limitation of PCR-based detection is its inability to distinguish between viable parasites and residual DNA originating from recent blood meals. Therefore, further studies, such as parasite isolation, cultivation, and experimental infection assays involving *Leishmania* and their suspected vectors, are necessary to evaluate their potential for transmission.

This study demonstrated the co-occurrence of *Leishmania* parasites and endosymbionts, exhibiting distinct patterns of *Wolbachia* and *Cardinium* infection. Additionally, it revealed the presence of double infections with *Wolbachia* and *Cardinium*. A research publication by Zhao et al. (2013) suggested that multiple infections of *Cardinium* and *Wolbachia* affect host age through a combination of CI shared in the spider mite (*Tetranychus phaselus*). Nevertheless, the function of *Cardinium* infection in arthropod fitness remains uncertain. In *Culicoides* biting midges, *Cardinium* infection in *C. imicola* did not affect the survival rate under laboratory conditions (Morag et al., 2013). The presence of *Wolbachia* supergroups A and B in sympatric infections was identified in *Leishmania*-positive samples. However, *Wolbachia* Supergroup F and co-infection with any strains were not identified in *Leishmania*-positive samples. Azpurua et al. (2010) demonstrated the occurrence of *Leishmania* and *Wolbachia* in *Lutzomyia trapidoi*. In contrast, previous reports of *Wolbachia*-positive sand flies and *Leishmania* DNA have demonstrated the absence of co-infection between the two organisms in sand flies (Lozano-Sardaneta et al., 2022). Although the impact of *Wolbachia* on pathogen development, including viruses, filarial nematodes, and malaria, has been examined in various insect species, there is currently no evidence of *Wolbachia* infection or a study of host-symbiont-pathogen interactions, particularly in *Leishmania* parasites and their bacteria in biting midges.

The present investigation demonstrates that endosymbiotic bacteria, including single infections with *Wolbachia* and *Cardinium*, as well as co-infection with both, are significantly associated with *Leishmania* DNA detection. We hypothesized that the presence of endosymbionts would be associated with negative detection of *Leishmania* DNA. However, both single and co-infections with *Wolbachia* and *Cardinium* were detected in samples that were either positive or negative for *Leishmania* DNA. These findings indicate no clear association between endosymbiont presence and *Leishmania* DNA detection in *Culicoides* spp. These findings highlight the complexity of tripartite associations among hosts, symbionts, and pathogens (Schinkel et al., 2024). Further experimental and longitudinal studies are required to clarify the mechanical basis of these relationships and to determine whether endosymbionts directly affect *Leishmania* development or transmission.

## 5 Conclusions

To the best of our knowledge, this is the first report of *Wolbachia* and *Cardinium* endosymbionts in field-collected *Culicoides* spp. from *Leishmania*-affected areas in Thailand. In this study, *Wolbachia* belonging to supergroups A, B, and F were identified, and putative strains including *w*Kerlac, *w*Beva_B, and *w*CauA were characterized. Additionally, *Cardinium* of groups A and C was detected. Co-occurrence of these bacterial endosymbionts with *Leishmania* parasites was also demonstrated. These findings provide further evidence for the tripartite interactions among hosts, symbionts, and pathogens, with potential implications for vector control strategies. However, the functional roles and properties of *Wolbachia* and *Cardinium* in influencing vector fitness and pathogen transmission require further investigation.

## Ethical approval

The study protocol was approved by the Animal Research Ethics Committee of Chulalongkorn University Animal Care and Use Protocol (CU-ACUP), Faculty of Medicine, Chulalongkorn University, Bangkok, Thailand (COA No. 2391008).

## CRediT authorship contribution statement

**Sakone Sunantaraporn:** Conceptualization, Methodology, Investigation, Data curation, Formal analysis, Validation, Writing – original draft, Writing – review & editing. **Pathamet Khositharattanakool:** Methodology, Investigation. **Puckavadee Somwang:** Methodology, Investigation. **Pranyu Leemingsawat:** Methodology, Validation. **Picha Pattrapruettada:** Methodology, Data curation. **Darlene Ariyaskul:** Methodology, Data curation. **Tinn Hongboontry:** Methodology, Data curation. **Chitchanok Cherdchoochart:** Methodology, Data curation. **Rungfar Boonserm:** Methodology, Investigation. **Thanapat Pataradool:** Methodology, Validation. **Padet Siriyasatien:** Conceptualization, Project administration, Supervision, Methodology, Data curation, formal analysis, Funding acquisition, Writing – review & editing.

## Statement on the use of AI-assisted technologies

During the preparation of this work the authors used Grammarly (https://www.grammarly.com/) to correct grammatical errors and improve readability. After using this tool, the authors reviewed and edited the content as needed and take full responsibility for the content of the published article.

## Funding

This research project was supported by 10.13039/501100002873Chulalongkorn University, the Second Century Fund (C2F).

## Declaration of competing interests

The authors declare that they have no known competing financial interests or personal relationships that could have influenced the work reported in this paper.

## Data Availability

All data generated or analyzed during this study are included in this published article and its supplementary files. The newly generated sequences were submitted to the GenBank database under the accession numbers PX884007-PX884058 (*Wolbachia*), PX845917-PX845968 (*Cardinium*), PX856299-PX856349 (*L. martiniquensis*), and PX856350-PX856357 (*L. orientalis*).
