# Supplementary material for: Molecular identification and characterization of Wolbachia and Cardinium with co-occurrence of Leishmania spp. in Culicoides biting midges (Diptera: Ceratopogonidae) from leishmaniasis-affected areas of Thailand
Source: Curr Res Parasitol Vector Borne Dis. 2026 May 16;9:100387. doi: 10.1016/j.crpvbd.2026.100387 (PMC13264255; doi:10.1016/j.crpvbd.2026.100387)
Supplement: Multimedia component 1 [file mmc1.pdf]

Table S1 Identification of *Wolbachia* in *Culicoides* spp. and corresponding BLAST results obtained from the GenBank database.

NO.	Code	*Culicoides* species	*Wolbachia* supergroup	Accession no.	%BLAST identity	Closest references sequence in GenBank
1	ANR3	*Culicoides oxystoma*	B	PX884007	99.83	*Kerria lacca* LIKBL39 (JQ837254)
2	ANR4	*Culicoides mahasarakhamense*	B	PX884008	100.00	*Kerria lacca* LIKBL39 (JQ837254)
3	MGSS13	*Culicoides mahasarakhamense*	B	PX884009	100.00	*Kerria lacca* LIKBL39 (JQ837254)
4	MGSS18	*Culicoides orientalis*	B	PX884010	96.66	*Chilo suppressalis* (HQ336511)
5	MGSS26	*Culicoides mahasarakhamense*	B	PX884011	99.83	*Kerria lacca* LIKBL39 (JQ837254)
6	MGSS28-26	*Culicoides guttifer*	B	PX884012	100.00	*Kerria lacca* LIKBL39 (JQ837254)
7	MGSS37	*Culicoides guttifer*	B	PX884013	100.00	*Kerria lacca* LIKBL39 (JQ837254)
8	SKSJ6	*Culicoides guttifer*	B	PX884014	98.45	*Acrogonia virescens* (DQ450156)
9	SKSJ8	*Culicoides guttifer*	B	PX884015	99.83	*Kerria lacca* LIKBL39 (JQ837254)
10	SKSJ28	*Culicoides huffi*	B	PX884016	100.00	*Kerria lacca* LIKBL39 (JQ837254)
11	T1SS46	*Culicoides peregrinus*	B	PX884017	99.83	*Kerria lacca* LIKBL39 (JQ837254)
12	T1SS48	*Culicoides peregrinus*	B	PX884018	99.83	*Kerria lacca* LIKBL39 (JQ837254)
13	T1SS49	*Culicoides peregrinus*	B	PX884019	99.66	*Kerria lacca* LIKBL39 (JQ837254)
14	T1SS59	*Culicoides peregrinus*	B	PX884020	100.00	*Kerria lacca* LIKBL39 (JQ837254)
15	T3C3	*Culicoides oxystoma*	B	PX884021	99.83	*Kerria lacca* LIKBL39 (JQ837254)
16	CRP93	*Culicoides orientalis*	B	PX884022	99.83	*Xanthorhoe designata* (OZ034794)
17	CRP99	*Culicoides jacobsoni*	B	PX884023	99.16	*Sesamia inferens* (KP822799)
18	CRP103	*Culicoides* (*Trithecoides*) sp.	B	PX884024	97.47	*Libythea celtis* (AB094388)
19	CRP113	*Culicoides orientalis*	B	PX884025	99.83	*Xanthorhoe designata* (OZ034794)
20	CRP140	*Culicoides tainanus*	B	PX884026	99.83	*Xanthorhoe designata* (OZ034794)
21	CRP141	*Culicoides insignipennis*	B	PX884027	97.47	*Libythea celtis* (AB094388)
22	CRP146	*Culicoides tainanus*	B	PX884028	99.83	*Xanthorhoe designata* (OZ034794)
23	CRP14	*Culicoides jacobsoni*	B	PX884029	98.10	*Acrogonia virescens* (DQ450156)
24	CRP29	*Culicoides malayae*	B	PX884030	99.16	*Kerria lacca* LIKBL39 (JQ837254)
25	CRP35	*Culicoides malayae*	B	PX884031	99.33	*Kerria lacca* LIKBL39 (JQ837254)
26	CRP48	*Culicoides tainanus*	B	PX884032	97.47	*Libythea celtis* (AB094388)
27	CRP64	*Culicoides orientalis*	B	PX884033	99.83	*Kerria lacca* LIKBL39 (JQ837254)
28	CRP84	*Culicoides orientalis*	B	PX884034	98.28	*Acrogonia virescens* (DQ450156)
29	LP80	*Culicoides oxystoma*	B	PX884035	99.83	*Xanthorhoe designata* (OZ034794)
30	LP129	*Culicoides actoni*	B	PX884036	99.83	*Xanthorhoe designata* (OZ034794)
31	LP128	*Culicoides peregrinus*	B	PX884037	99.83	*Xanthorhoe designata* (OZ034794)
32	LP98	*Culicoides guttifer*	B	PX884038	99.83	*Xanthorhoe designata* (OZ034794)
33	LP100	*Culicoides huffi*	B	PX884039	99.83	*Xanthorhoe designata* (OZ034794)
34	LP101	*Culicoides insignipennis*	B	PX884040	99.83	*Xanthorhoe designata* (OZ034794)
35	LP54	*Culicoides shortti*	B	PX884041	99.83	*Kerria lacca* LIKBL39 (JQ837254)
36	LP82	*Culicoides mahasarakhamense*	B	PX884042	99.83	*Kerria lacca* LIKBL39 (JQ837254)
37	SKSJ1	*Culicoides guttifer*	A	PX884043	100.00	*Ephestia cautella* (AB024571)
38	CRP90	*Culicoides oxystoma*	A	PX884044	99.67	*Armigeres subalbatus* (MW717996)
39	CRP107	*Culicoides tainanus*	A	PX884045	99.67	*Armigeres subalbatus* (MW717996)
40	LP1	*Culicoides palpifer*	A	PX884046	98.52	*Armigeres obturbansm* (KJ140131)
41	LP150	*Culicoides innoxius*	A	PX884047	99.67	*Armigeres subalbatus* (MW717996)
42	LP151	*Culicoides actoni*	A	PX884048	98.52	*Armigeres obturbansm* (KJ140131)
43	LP109	*Culicoides jacobsoni*	A	PX884049	99.67	*Armigeres subalbatus* (MW717996)
44	LP160	*Culicoides peregrinus*	A	PX884050	97.54	*Armigeres obturbansm* (KJ140131)

NO.	Code	*Culicoides* species	*Wolbachia* supergroup	Accession no.	%BLAST identity	Closest references sequence in GenBank
45	LP164	*Culicoides fulvus*	A	PX884051	97.54	*Armigeres obturbansm* (KJ140131)
46	LP220	*Culicoides shortti*	A	PX884052	98.52	*Armigeres obturbansm* (KJ140131)
47	LP79	*Culicoides oxystoma*	F	PX884053	91.43	*Ctenocephalides felis* (CP116768)
48	LP91	*Culicoides sumatrae*	F	PX884054	92.06	*Ctenocephalides felis* (CP116768)
49	LP218	*Culicoides palpifer*	F	PX884055	92.52	*Ctenocephalides felis* (CP116768)
50	MGSS28-28	*Culicoides guttifer*	F	PX884056	91.43	*Ctenocephalides felis* (CP116768)
51	MGSS29	*Culicoides guttifer*	F	PX884057	92.06	*Ctenocephalides felis* (CP116768)
52	T1SS27	*Culicoides peregrinus*	F	PX884058	92.52	*Ctenocephalides felis* (CP116768)

Table S2 Identification of *Cardinium* in *Culicoides* spp. and corresponding BLAST results obtained from the GenBank database.

No.	Code	*Culicoides* species	*Cardinium* group	Accession no.	%BLAST identity	Closest references sequence in GenBank
1	CRP28	*Culicoides liui*	A	PX845917	98.82	*Microzetorchestes emeryi* (MG889459)
2	CRP29	*Culicoides malayae*	C	PX845918	99.83	*Culicoides williwilli* (KR026922)
3	CRP35	*Culicoides malayae*	C	PX845919	97.98	*Culicoides williwilli* (KR026922)
4	CRP44	*Culicoides tainanus*	C	PX845920	97.98	*Culicoides williwilli* (KR026922)
5	CRP46	*Culicoides jacobsoni*	C	PX845921	99.50	*Culicoides williwilli* (KR026922)
6	CRP51	*Culicoides* (*Trithecoides*) sp.	C	PX845922	99.83	*Culicoides williwilli* (KR026922)
7	CRP60	*Culicoides liui*	C	PX845923	98.99	*Culicoides williwilli* (KR026922)
8	CRP64	*Culicoides orientalis*	C	PX845924	98.99	*Culicoides williwilli* (KR026922)
9	LP28	*Culicoides guttifer*	C	PX845925	99.50	*Culicoides williwilli* (KR026922)
10	LP29	*Culicoides guttifer*	C	PX845926	99.33	*Culicoides williwilli* (KR026922)
11	LP30	*Culicoides mahasarakhamense*	C	PX845927	99.16	*Culicoides williwilli* (KR026922)
12	LP54	*Culicoides shortti*	C	PX845928	99.83	*Culicoides williwilli* (KR026922)
13	LP55	*Culicoides orientalis*	C	PX845929	99.16	*Culicoides williwilli* (KR026922)
14	LP60	*Culicoides mahasarakhamense*	C	PX845930	99.83	*Culicoides williwilli* (KR026922)
15	LP80	*Culicoides oxystoma*	A	PX845931	98.82	*Microzetorchestes emeryi* (MG889459)
16	LP98	*Culicoides guttifer*	C	PX845932	99.50	*Culicoides williwilli* (KR026922)
17	LP123	*Culicoides tenuipalpis*	C	PX845933	99.33	*Culicoides williwilli* (KR026922)
18	LP125	*Culicoides oxystoma*	C	PX845934	99.50	*Culicoides williwilli* (KR026922)
19	LP128	*Culicoides peregrinus*	A	PX845935	98.82	*Microzetorchestes emeryi* (MG889459)
20	LP129	*Culicoides actoni*	A	PX845936	98.99	*Microzetorchestes emeryi* (MG889459)
21	LP146	*Culicoides mahasarakhamense*	C	PX845937	99.33	*Culicoides williwilli* (KR026922)
22	LP152	*Culicoides oxystoma*	C	PX845938	99.50	*Culicoides williwilli* (KR026922)
23	LP160	*Culicoides peregrinus*	A	PX845939	98.82	*Microzetorchestes emeryi* (MG889459)
24	LP164	*Culicoides fulvus*	A	PX845940	98.65	*Microzetorchestes emeryi* (MG889459)
25	LP169	*Culicoides orientalis*	A	PX845941	98.99	*Microzetorchestes emeryi* (MG889459)
26	LP171	*Culicoides innoxius*	A	PX845942	98.82	*Microzetorchestes emeryi* (MG889459)
27	LP172	*Culicoides orientalis*	A	PX845943	98.82	*Microzetorchestes emeryi* (MG889459)
28	LP218	*Culicoides palpifer*	C	PX845944	99.83	*Culicoides williwilli* (KR026922)
29	LP220	*Culicoides shortti*	A	PX845945	98.82	*Microzetorchestes emeryi* (MG889459)
30	CNR12	*Culicoides mahasarakhamense*	C	PX845946	99.50	*Culicoides williwilli* (KR026922)
31	MGSS2	*Culicoides guttifer*	C	PX845947	99.33	*Culicoides williwilli* (KR026922)
32	MGSS10	*Culicoides guttifer*	C	PX845948	99.33	*Culicoides williwilli* (KR026922)
33	MGSS11	*Culicoides guttifer*	C	PX845949	99.50	*Culicoides williwilli* (KR026922)
34	MGSS12	*Culicoides guttifer*	C	PX845950	99.33	*Culicoides williwilli* (KR026922)
35	MGSS13	*Culicoides mahasarakhamense*	C	PX845951	99.83	*Culicoides williwilli* (KR026922)
36	MGSS20	*Culicoides guttifer*	C	PX845952	99.33	*Culicoides williwilli* (KR026922)
37	MGSS26	*Culicoides guttifer*	C	PX845953	99.83	*Culicoides williwilli* (KR026922)
38	SKSJ2	*Culicoides guttifer*	C	PX845954	99.33	*Culicoides williwilli* (KR026922)
39	SKSJ4	*Culicoides mahasarakhamense*	C	PX845955	99.33	*Culicoides williwilli* (KR026922)
40	T1PS1	*Culicoides peregrinus*	C	PX845956	99.50	*Culicoides williwilli* (KR026922)
41	T1PS14	*Culicoides mahasarakhamense*	C	PX845957	99.33	*Culicoides williwilli* (KR026922)
42	T1PS17	*Culicoides mahasarakhamense*	C	PX845958	99.50	*Culicoides williwilli* (KR026922)
43	T1PS22	*Culicoides guttifer*	C	PX845959	99.50	*Culicoides williwilli* (KR026922)
44	T1PS32	*Culicoides mahasarakhamense*	C	PX845960	99.66	*Culicoides williwilli* (KR026922)

No.	Code	*Culicoides* species	*Cardinium* group	Accession no.	%BLAST identity	Closest references sequence in GenBank
45	T1PS37	*Culicoides mahasarakhamense*	C	PX845961	99.83	*Culicoides williwilli* (KR026922)
46	T1SS15	*Culicoides peregrinus*	A	PX845962	98.99	*Microzetorchestes emeryi* (MG889459)
47	T1SS43	*Culicoides mahasarakhamense*	C	PX845963	99.83	*Culicoides williwilli* (KR026922)
48	T1SS61	*Culicoides mahasarakhamense*	C	PX845964	99.66	*Culicoides williwilli* (KR026922)
49	TBY8	*Culicoides mahasarakhamense*	C	PX845965	99.83	*Culicoides williwilli* (KR026922)
50	TBY11	*Culicoides guttifer*	C	PX845966	99.50	*Culicoides williwilli* (KR026922)
51	TBY12	*Culicoides guttifer*	C	PX845967	99.16	*Culicoides williwilli* (KR026922)
52	TBY13	*Culicoides guttifer*	C	PX845968	99.50	*Culicoides williwilli* (KR026922)

Table S3 Study areas and *Culicoides* spp. detected with *Leishmania* parasites and endosymbiont infection.

No.	Location	Sample ID	*Culicoides* species	*Leishmania* spp.	*Leishmania* spp. (Co-infection)	Endosymbionts infection
1	Songkhla (SK2)	T1SS11	*C. peregrinus*	*L. martiniquensis*	-	ND
2	Songkhla (SK2)	T1SS19	*C. oxystoma*	*L. martiniquensis*	-	ND
3	Songkhla (SK2)	T1SS33	*C. peregrinus*	*L. martiniquensis*	-	*Wolbachhia*
4	Songkhla (SK2)	T1SS38	*C. oxystoma*	*L. martiniquensis*	-	*Wolbachhia*
5	Songkhla (SK2)	T1SS45	*C. peregrinus*	*L. martiniquensis*	-	*Wolbachhia*
6	Songkhla (SK2)	T1SS46	*C. peregrinus*	*L. martiniquensis*	-	*Wolbachhia*
7	Songkhla (SK2)	T1SS49	*C. peregrinus*	*L. martiniquensis*	-	*Wolbachhia*
8	Songkhla (SK2)	T1SS50	*C. peregrinus*	*L. martiniquensis*	-	*Wolbachhia*
9	Songkhla (SK2)	T1SS53	*C. peregrinus*	*L. martiniquensis*	-	ND
10	Songkhla (SK3)	SKSJ1	*C. guttifer*	*L. martiniquensis*	-	*Wolbachhia* + *Cardinium*
11	Songkhla (SK3)	SKSJ2	*C. guttifer*	*L. martiniquensis*	-	*Cardinium*
12	Songkhla (SK3)	SKSJ4	*C. mahasarakhamense*	*L. martiniquensis*	-	*Cardinium*
13	Songkhla (SK3)	SKSJ11	*C. mahasarakhamense*	*L. martiniquensis*	-	*Wolbachhia* + *Cardinium*
14	Songkhla (SK3)	SKSJ12	*C. guttifer*	*L. martiniquensis*	-	*Cardinium*
15	Lampang	LP25	*C. mahasarakhamense*	*L. martiniquensis*	-	*Wolbachhia* + *Cardinium*
16	Lampang	LP28	*C. guttifer*	*L. martiniquensis*	-	*Wolbachhia* + *Cardinium*
17	Lampang	LP29	*C. guttifer*	*L. martiniquensis*	-	*Wolbachhia* + *Cardinium*
18	Lampang	LP30	*C. mahasarakhamense*	*L. martiniquensis*	-	*Wolbachhia* + *Cardinium*
19	Lampang	LP35	*C. mahasarakhamense*	*L. martiniquensis*	-	*Cardinium*
20	Lampang	LP54	*C. shortti*	*L. martiniquensis*	-	*Wolbachhia* + *Cardinium*
21	Lampang	LP56	*C. oxystoma*	*L. martiniquensis*	-	*Cardinium*
22	Lampang	LP60	*C. mahasarakhamense*	*L. martiniquensis*	-	*Cardinium*
23	Lampang	LP97	*C. mahasarakhamense*	*L. martiniquensis*	-	*Wolbachhia* + *Cardinium*
24	Lampang	LP98	*C. guttifer*	-	*L. martiniquensis* + *L. orientalis*	*Wolbachhia* + *Cardinium*
25	Lampang	LP118	*C. oxystoma*	*L. martiniquensis*	-	*Wolbachhia*
26	Lampang	LP129	*C. actoni*	*L. martiniquensis*	-	*Wolbachhia* + *Cardinium*
27	Lampang	LP154	*C. shortti*	-	*L. martiniquensis* + *L. orientalis*	*Wolbachhia*
28	Lampang	LP160	*C. peregrinus*	-	*L. martiniquensis* + *L. orientalis*	*Wolbachhia* + *Cardinium*
29	Lampang	LP161	*C. insignipennis*	*L. martiniquensis*	-	*Wolbachhia*
30	Lampang	LP162	*C. shortti*	*L. martiniquensis*	-	*Wolbachhia*
31	Lampang	LP163	*C. peregrinus*	*L. martiniquensis*	-	*Wolbachhia* + *Cardinium*
32	Lampang	LP164	*C. fulvus*	-	*L. martiniquensis* + *L. orientalis*	*Wolbachhia* + *Cardinium*
33	Lampang	LP168	*C. sumatrae*	*L. martiniquensis*	-	*Wolbachhia* + *Cardinium*
34	Lampang	LP171	*C. innoxius*	*L. martiniquensis*	-	*Wolbachhia* + *Cardinium*
35	Lampang	LP219	*C. shortti*	*L. martiniquensis*	-	*Wolbachhia*
36	Lampang	LP170	*C. orientalis*	*L. orientalis*	-	*Wolbachhia* + *Cardinium*
37	Lampang	LP172	*C. orientalis*	*L. orientalis*	-	*Wolbachhia* + *Cardinium*
38	Lampang	LP220	*C. shortti*	*L. martiniquensis*	-	*Wolbachhia* + *Cardinium*
39	Chiang Rai	CRP7	*C. insignipennis*	*L. martiniquensis*	-	*Wolbachhia*
40	Chiang Rai	CRP9	*C. orientalis*	*L. martiniquensis*	-	ND
41	Chiang Rai	CRP24	*C. liui*	*L. martiniquensis*	-	*Wolbachhia*
42	Chiang Rai	CRP28	*C. liui*	*L. martiniquensis*	-	*Wolbachhia* + *Cardinium*
43	Chiang Rai	CRP53	*C. jacobsoni*	*L. martiniquensis*	-	ND
44	Chiang Rai	CRP68	*C.* (*Trithecoides*) sp.	*L. martiniquensis*	-	ND
45	Chiang Rai	CRP74	*C. huffi*	*L. martiniquensis*	-	*Wolbachhia*
46	Chiang Rai	CRP77	*C. tainanus*	*L. martiniquensis*	-	*Wolbachhia*
47	Chiang Rai	CRP84	*C. orientalis*	*L. martiniquensis*	-	*Wolbachhia*

No.	Location	Sample ID	*Culicoides* species	*Leishmania* sp.	*Leishmania* sp. (Co-infection)	Endosymbionts infection
48	Chiang Rai	CRP86	*C. orientalis*	*L. martiniquensis*	-	*Wolbachhia* + *Cardinium*
49	Chiang Rai	CRP101	*C. jacobsoni*	*L. martiniquensis*	-	ND
50	Chiang Rai	CRP108	*C. jacobsoni*	-	*L. martiniquensis*/*L. orientalis*	ND
51	Chiang Rai	CRP141	*C. insignipennis*	*L. martiniquensis*	-	*Wolbachhia*
52	Chiang Rai	CRP143	*C. tainanus*	-	*L. martiniquensis*/*L. orientalis*	ND
53	Chiang Rai	CRP146	*C. tainanus*	*L. martiniquensis*	-	*Wolbachhia*
